# Supplementary material for: Long-read transcript sequencing identifies differential isoform expression in the entorhinal cortex in a transgenic model of tau pathology
Source: Nat Commun. 2024 Aug 2;15:6458. doi: 10.1038/s41467-024-50486-8 (PMC11297290; doi:10.1038/s41467-024-50486-8)

Supplementary Figures

Supplementary Figure 1	Global transcript characteristics between WT and TG mice
Supplementary Figure 2	Presence of human *MAPT* transgene in TG mice
Supplementary Figure 3	Correlation of RNA-Seq and Iso-Seq gene expression
Supplementary Figure 4	Rarefaction curve to determine expression threshold
Supplementary Figure 5	Relationship between number of isoforms and other features
Supplementary Figure 6	Protein predictions - collapsing long-read transcripts by ORF
Supplementary Figure 7	Characterisation of ES and A5'A3' events in AD-associated genes
Supplementary Figure 8	Characterisation of IR in AD-associated genes
Supplementary Figure 9	Characterization of cryptic exons in AD-associated genes
Supplementary Figure 10	Top 10 differentially expressed transcripts (genotype)
Supplementary Figure 11	Differential transcript usage
Supplementary Figure 12	Increased abundance of transcripts characterized with IR
Supplementary Figure 13	Increased abundance of transcripts characterized with NMD
Supplementary Figure 14	Immunohistochemistry staining of Trem2, Iba1 and tau
Supplementary Figure 15	Further characterization of *Trem2*
Supplementary Figure 16	Dominant *Clu/CLU* isoforms in rTg4510 and human AD cortex
Supplementary Figure 17	Dominant *Bin1/BIN1* isoforms in rTg4510 and human AD cortex
Supplementary Figure 18	FANS gating strategy
Supplementary Figure 19	Uncropped scan from immunohistochemistry staining

Supplementary Figure 1: Global transcript characteristics between WT and TG mice. Shown are box-plots of the **(A)** isoform length and the **(B)** exon diversity of the isoforms commonly ("Both") and uniquely detected in WT and TG mice from the whole transcriptome PacBio Iso-Seq dataset. There was no significant difference between groups for any measure.

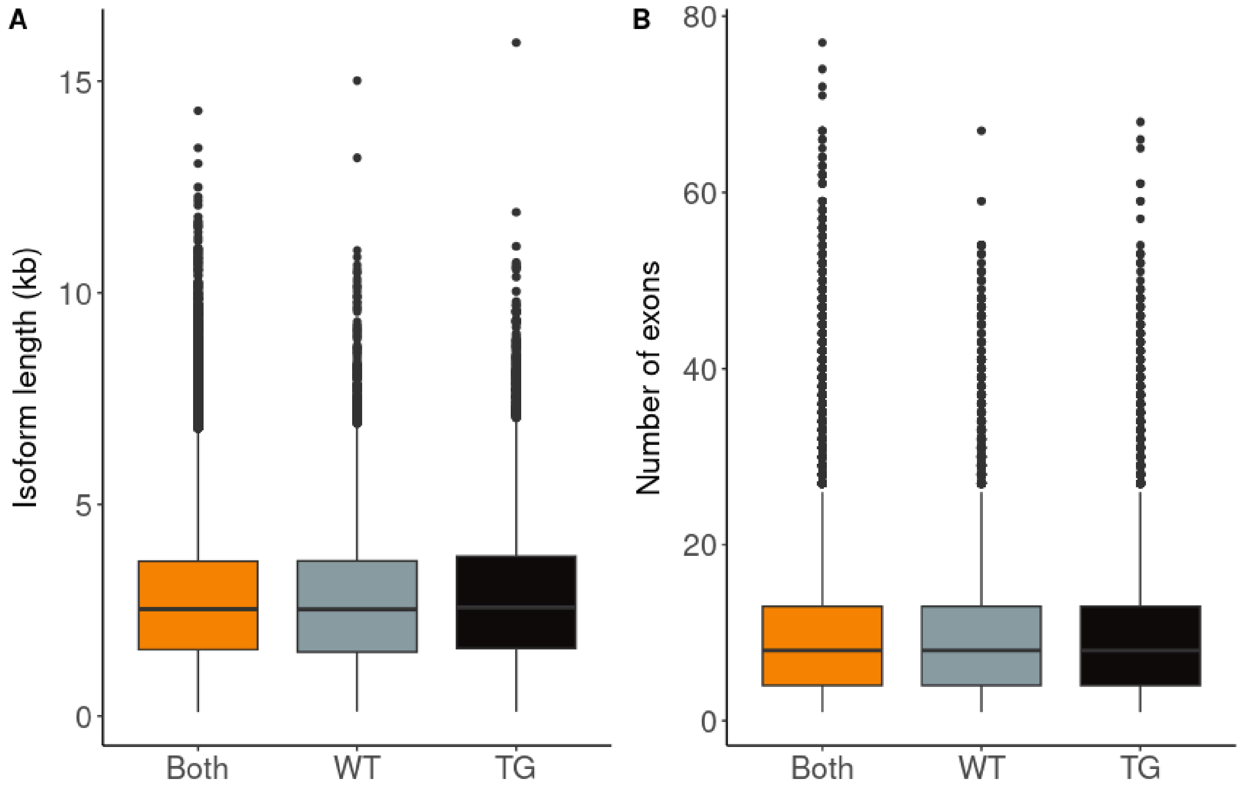

Supplementary Figure 2: Human-specific *MAPT* sequences were only present in transgenic mice.
Shown are scatter plots of the proportion of full-length reads that were mapped to human-specific *MAPT* and mouse-specific *Mapt* sequences in the **(A)** whole transcriptome PacBio Iso-Seq, **(B)** targeted PacBio Iso-Seq, and **(C)** targeted ONT datasets. Red and gray dots refer to TG and WT samples, and dotted lines represent the mean paths across age.

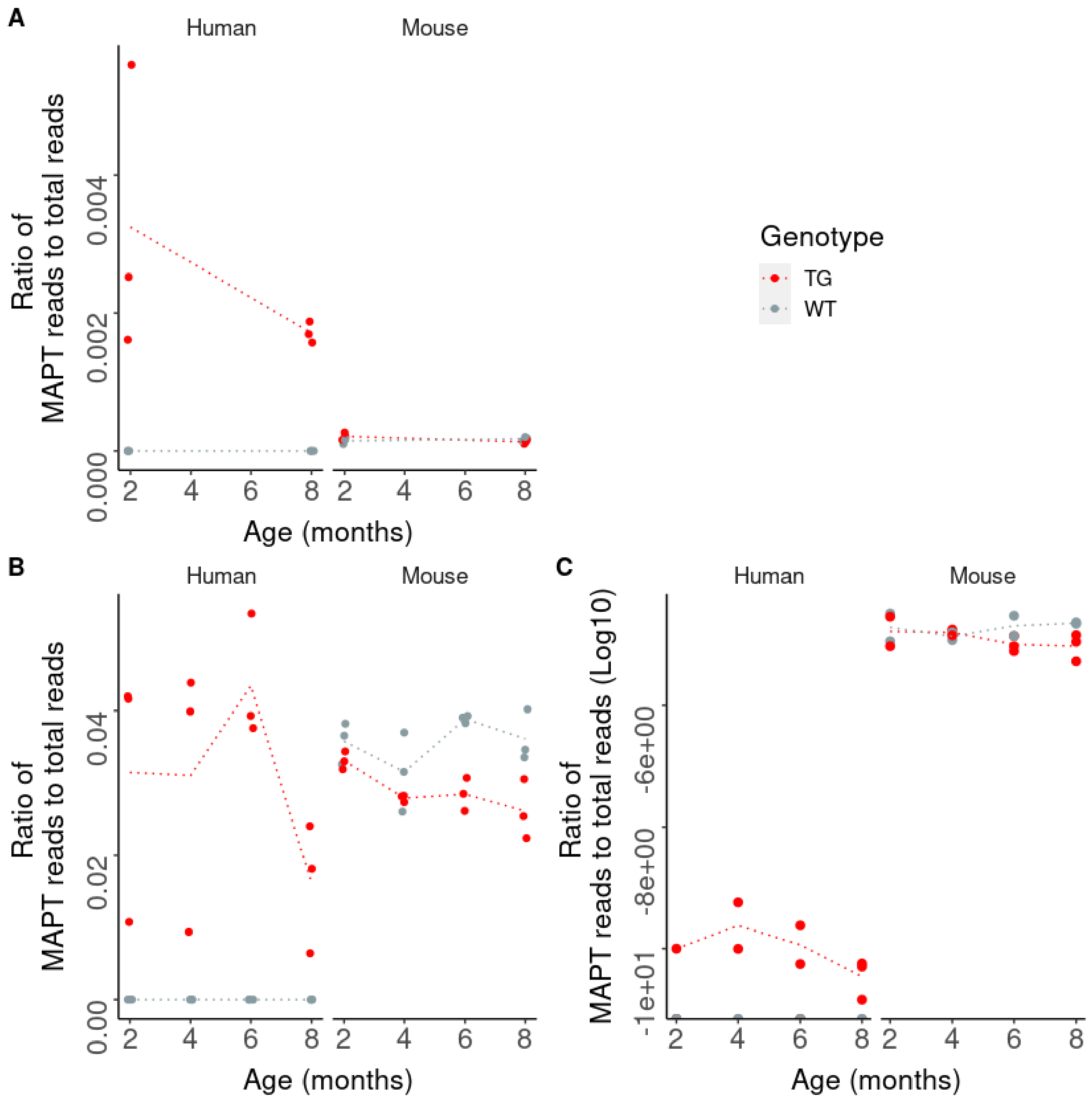

Supplementary Figure 3: Concordant gene expression differences observed between whole transcriptome Iso-Seq and RNA-Seq datasets.
Shown is a strong positive correlation (corr = 0.60, P = 5.17 x 10^{-224}) of differentially expressed gene (DEG) effect sizes (log_2 fold change between WT and TG at 8 months) derived from Iso-Seq full-length reads (whole transcriptome Iso-Seq dataset) and short-read RNA-Seq reads (RNA-Seq dataset) used as a proxy of gene expression in *DESeq2*. Each dot represents a gene that is differentially expressed between WT and TG mice with the progression of tau pathology (pathology effect) in the RNA-Seq (n = 59) dataset (Castanho et al. 2020), following alignment to transcript annotations derived from our Iso-Seq data.

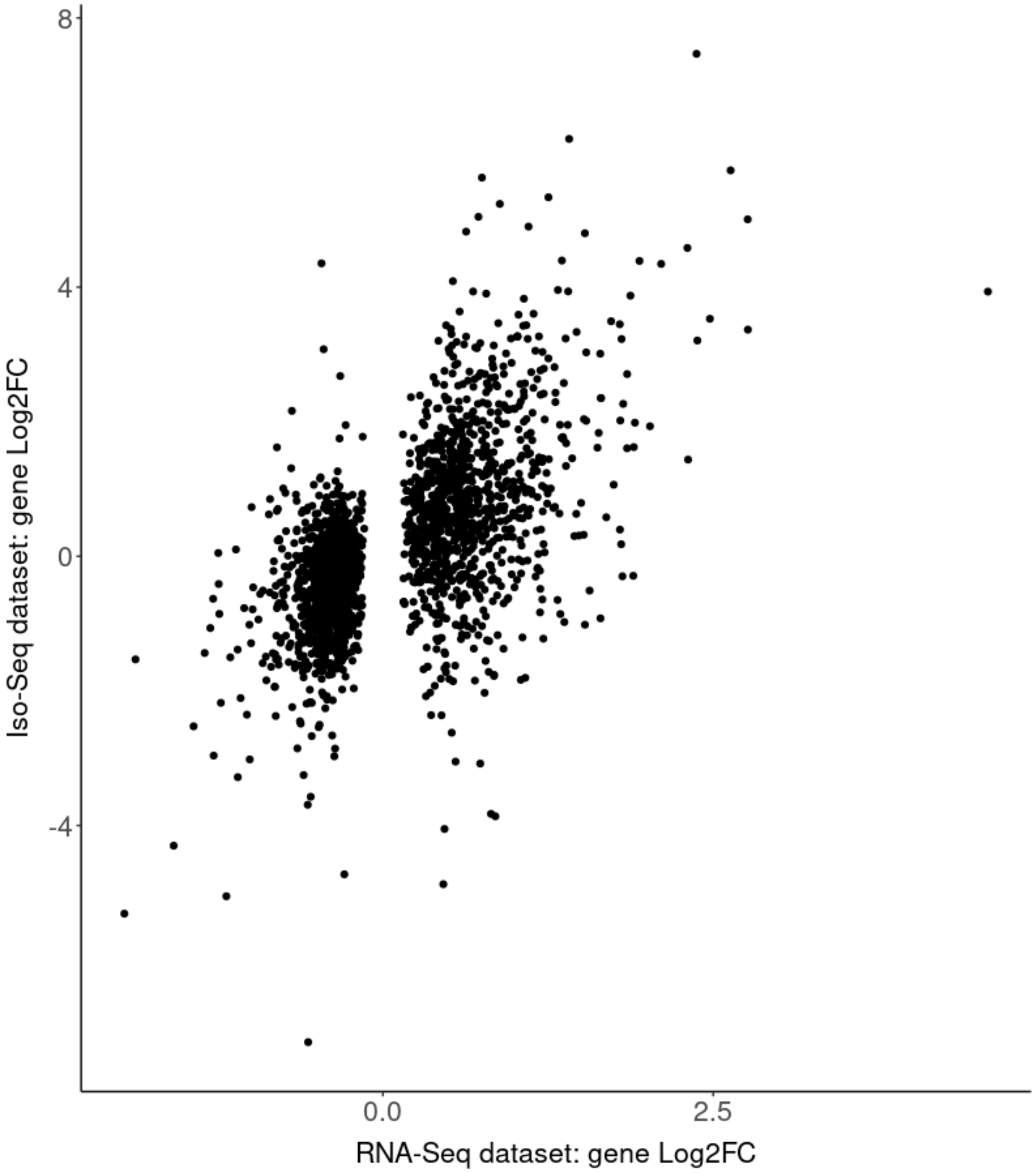

Supplementary Figure 4: Rarefaction curve to determine expression threshold for filtering targeted datasets.

Shown is a rarefaction curve of the percentage of transcripts retained after applying stepwise sample and read-count thresholds. For example, the red data points on the left-to-right X-axis represent the percentage of transcripts (associated to the 20 target genes from merged targeted ONT and Iso-Seq datasets) retained after applying thresholds of (total) full-length reads ≥ 2, 3, …10, 15, and 20 reads across any 2 samples. By identifying the point at which all the curves start to converge, we established a minimum expression threshold of 10 reads across at least 5 samples for filtering rare transcripts in our targeted datasets.

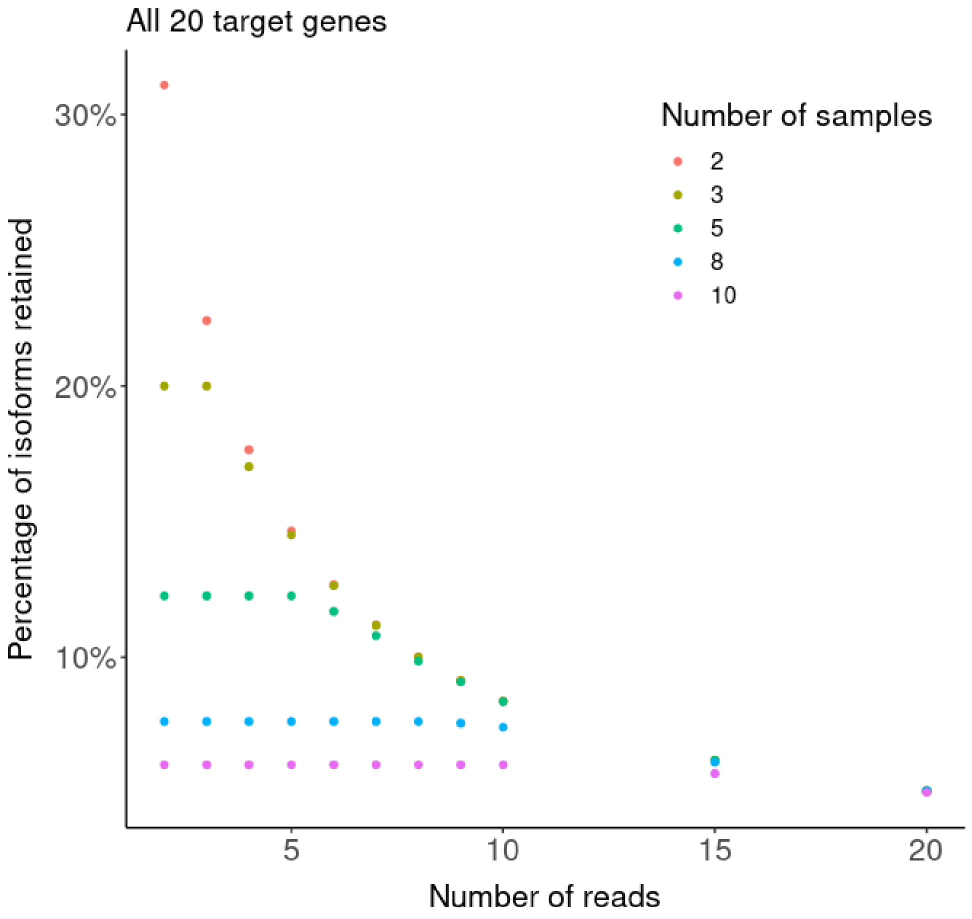

Supplementary Figure 5: Relationship between the number of isoforms and other features in targeted ONT dataset.

Shown are scatter plots of the number of detected isoforms against **(A)** gene length, **(B)** the number of exons, and **(C)** the normalized count as a proxy of gene expression. Each dot refers to a target gene from the targeted ONT dataset. The gene length and the number of exons (maximum number) are extracted from reference mouse annotations (mm10, GENCODE). **(D)** Distribution of isoforms detected by abundance across the target genes from the targeted ONT dataset. Each row refers to an isoform.

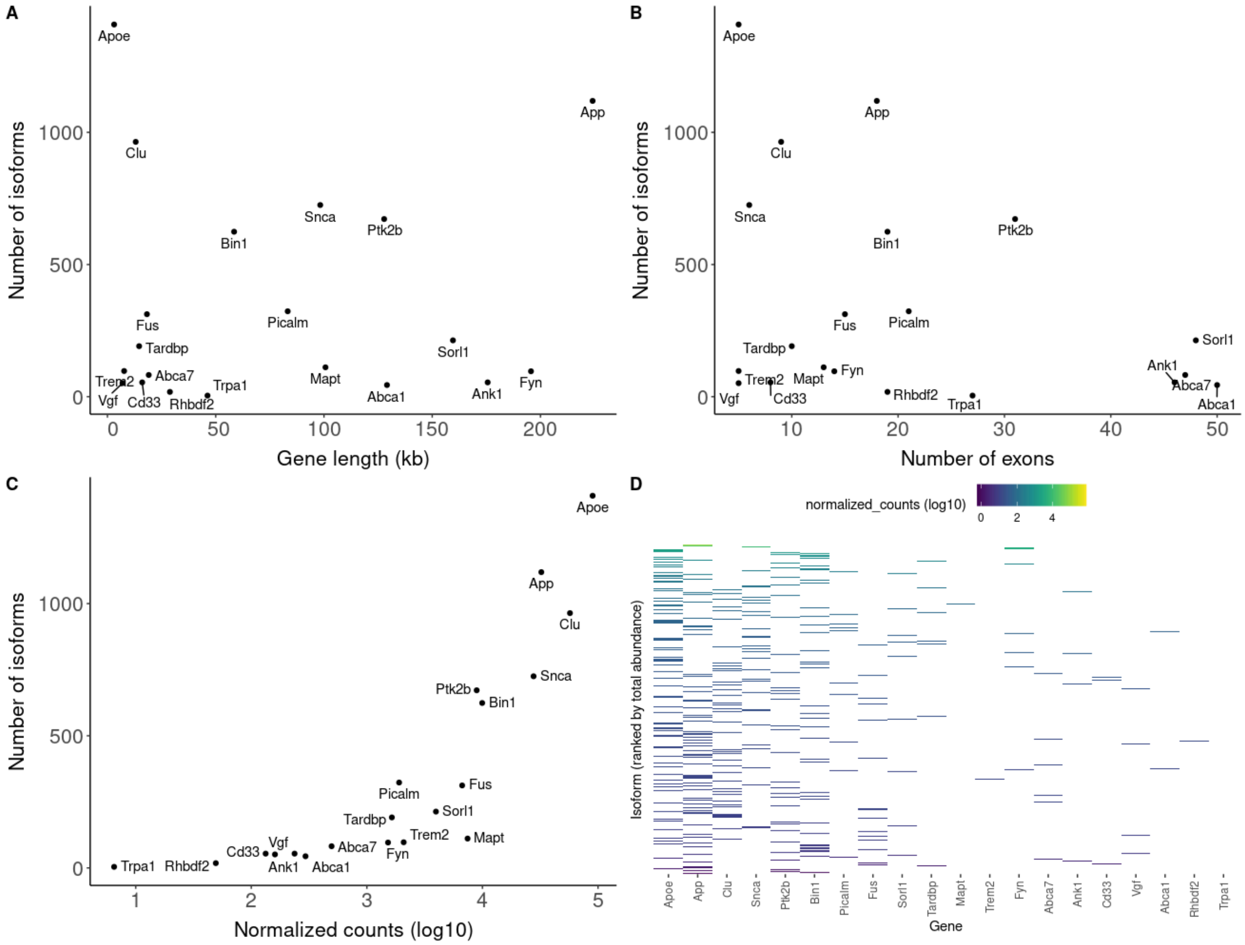

Supplementary Figure 6: Collapsing long-read transcripts in rTg4510 targeted transcriptome by open reading frames. Shown are **(A)** a scatter plot of the number of RNA transcripts to the number of protein isoforms with unique open reading frames (ORF) and coding sequence (CDS) – detected in the rTg4510 targeted dataset across the 20 AD-associated genes, **(B)** the number of “redundant” transcripts collapsed classified using *SQANTI3* structural and sub-categories, **(C)** visualization tracks of LR.Trem2.54 transcript and other minor *Trem2* transcripts that have the same ORF, and **(D)** visualization tracks of the **(i)** representative Trem2 transcripts that have unique ORFs, and **(ii)** the corresponding ORF. The RNA transcripts in the tracks are coloured by structural category (FSM – blue, NIC – red, NNC – pink) and the RNA isoforms with unique ORFs are coloured by coding potential (protein-coding – green, non protein-coding – red). FSM – Full Splice Match, NIC – Novel in Catalog, NNC – Novel Not in Catalog.

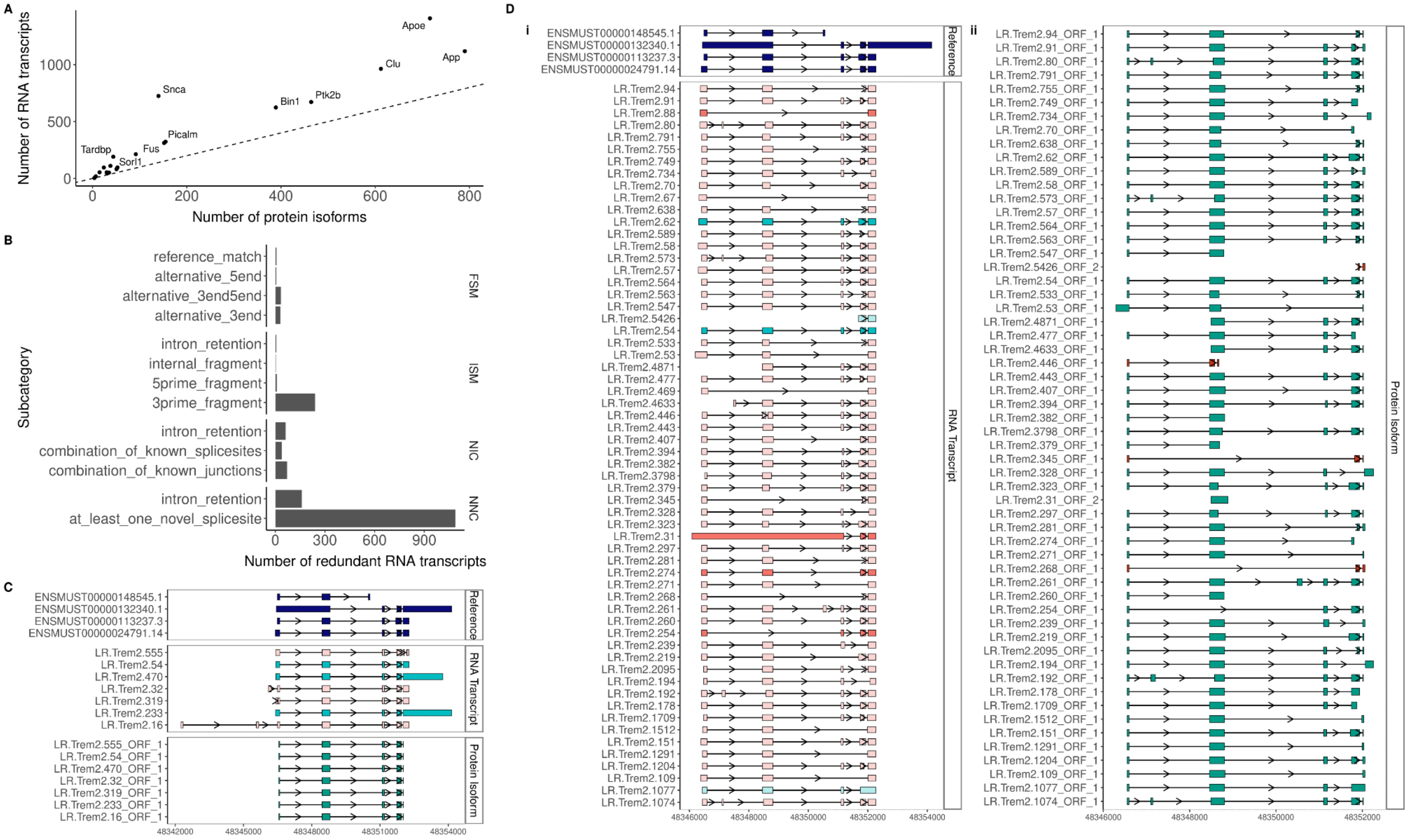

Supplementary Figure 7: Usage of alternative splice sites and exon skipping events are commonly observed in genes associated with AD.

Shown are bar-plots of **(A)** the number of isoforms classified with alternative 5' and 3' splice sites, and **(B)** the number of isoforms with exon-skipping events. Bar-plots were generated from *FICLE.* Of note, isoforms can be classified multiple times with different alternative splice sites. Also shown are examples of **(C)** known (FSM, ISM) and novel (NNC) isoforms annotated to *App* and **(D)** *Bin1*, characterized by no (green) and extensive (red) exon skipping events. Examples were found using *FICLE* and visualized using *ggtranscript*. FSM – Full Splice Match, ISM – Incomplete Splice Match, NIC – Novel in Catalog, NNC – Novel Not in Catalog

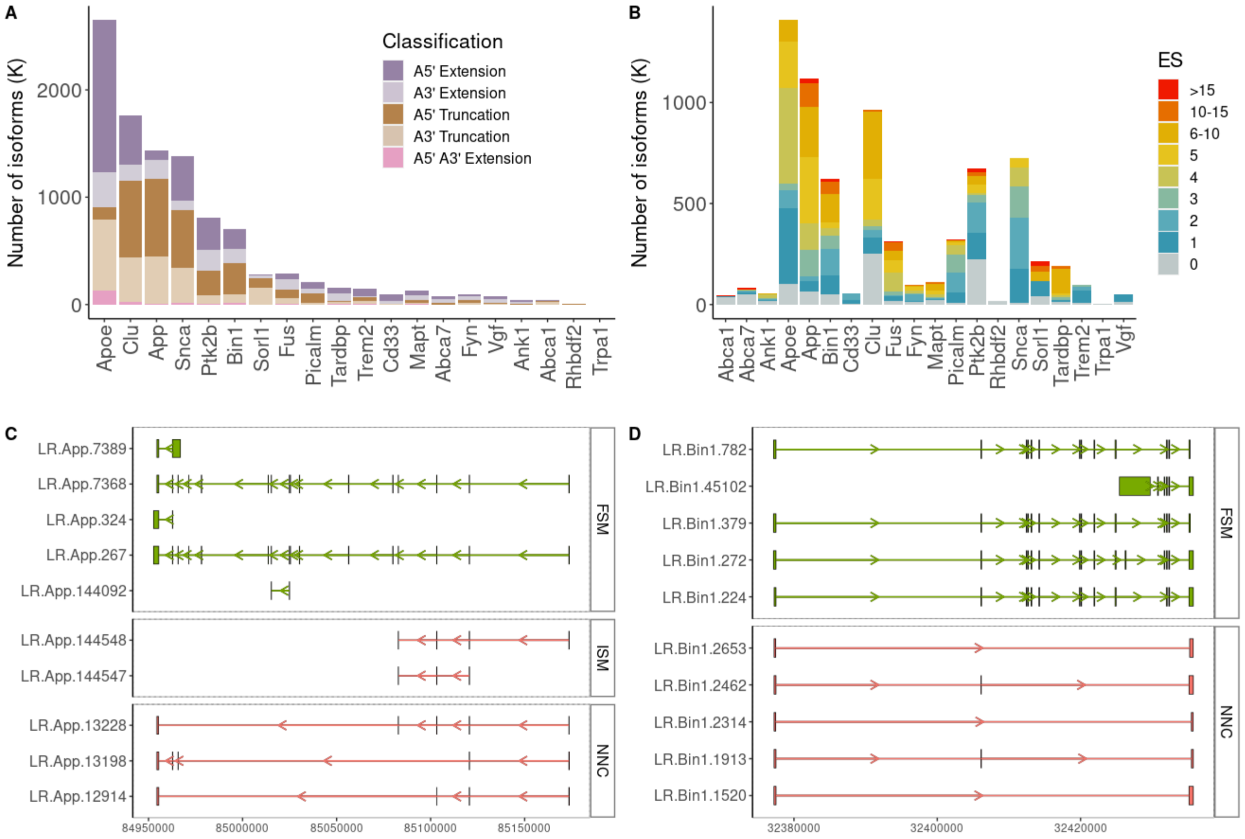

Supplementary Figure 8: Intron retention events, although less frequently detected than exon skipping, are also observed in genes associated with AD.

Shown are **(A)** reference *Cd33* transcripts and a novel isoform characterized by two distinct intron retention events, identified using *FICLE.* **(B)** Bar-plot of the number of isoforms with intron retention (IR) events, and the **(C)** number of isoforms with IR events spanning across multiple exons. Plots were generated using *FICLE*. **(D)** A box-plot of the expression of transcripts with 0, 1 and 2 intron retention events. **(E)** Visualization of reference *Tardbp* transcripts and isoforms with IR events spanning across multiple exons, noting the complexity of the 3'end of the gene body. Examples were found using *FICLE* and visualized using *ggtranscript*.

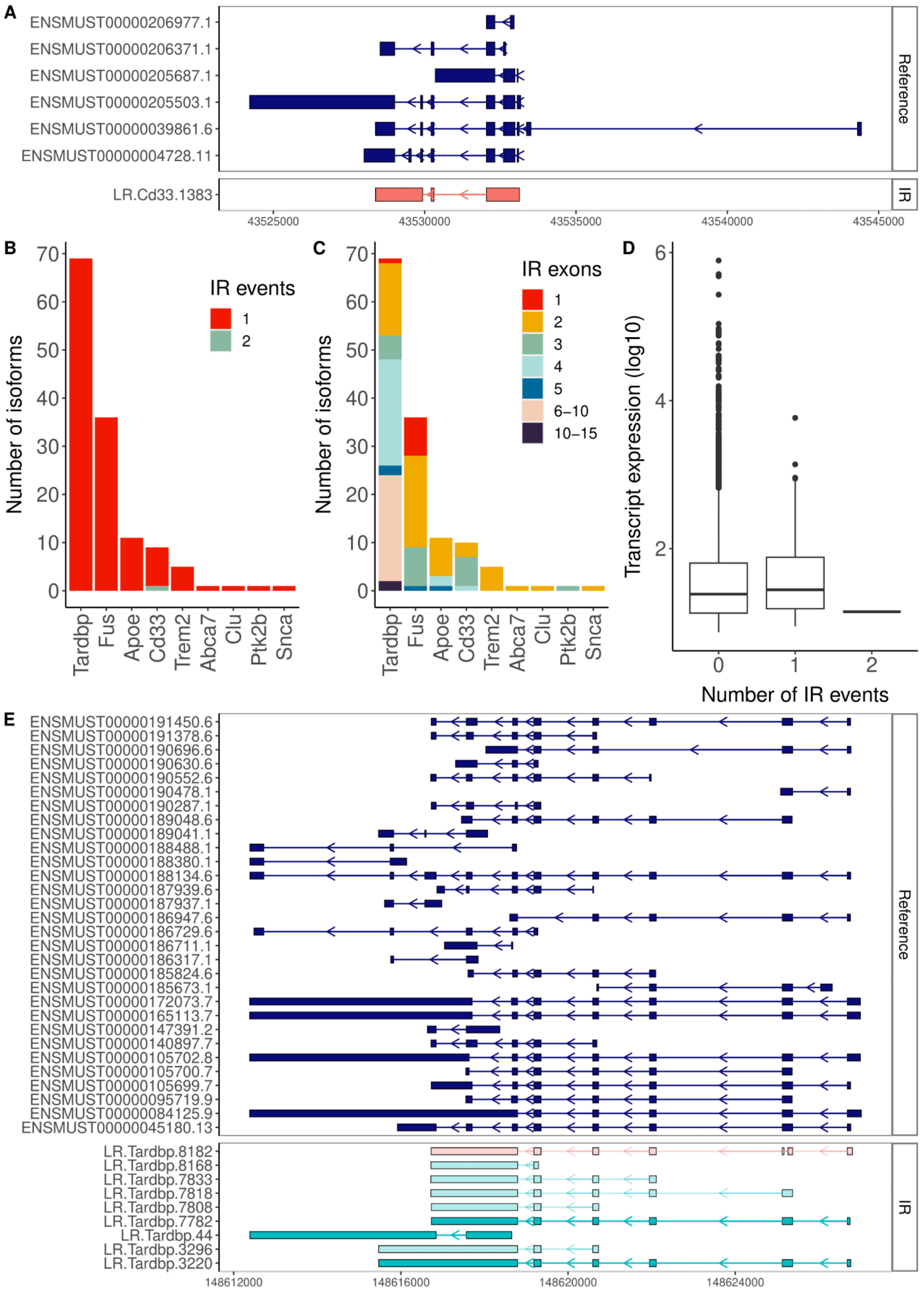

Supplementary Figure 9: Characterization of cryptic exons in AD-associated genes. Shown are track visualization of the transcript structure and respective predicted open reading frame of the reference transcripts and cryptic-exon-containing transcripts annotated to **(A)** *Apoe*, **(B)** *Bin1,* **(C)** *Clu,* **(D)** *Trem2* and **(E)** *Snca.* The reference transcripts are coloured in dark blue and paneled as “Reference”, and the cryptic-exon-containing transcripts identified from the targeted transcriptome datasets are pink with the predicted open reading frame (ORF) as gray and paneled either as “not NMD”, “NMD” or “non-coding”. The novel exons are marked out in the green box. The known transcripts (blue) and respective ORFs are also shown for *Apoe* and *Clu* for comparison. **(F)** Box-plot of the total normalization counts (ONT full-length reads) of the transcripts characterized with cryptic exons in WT and TG mice across age.

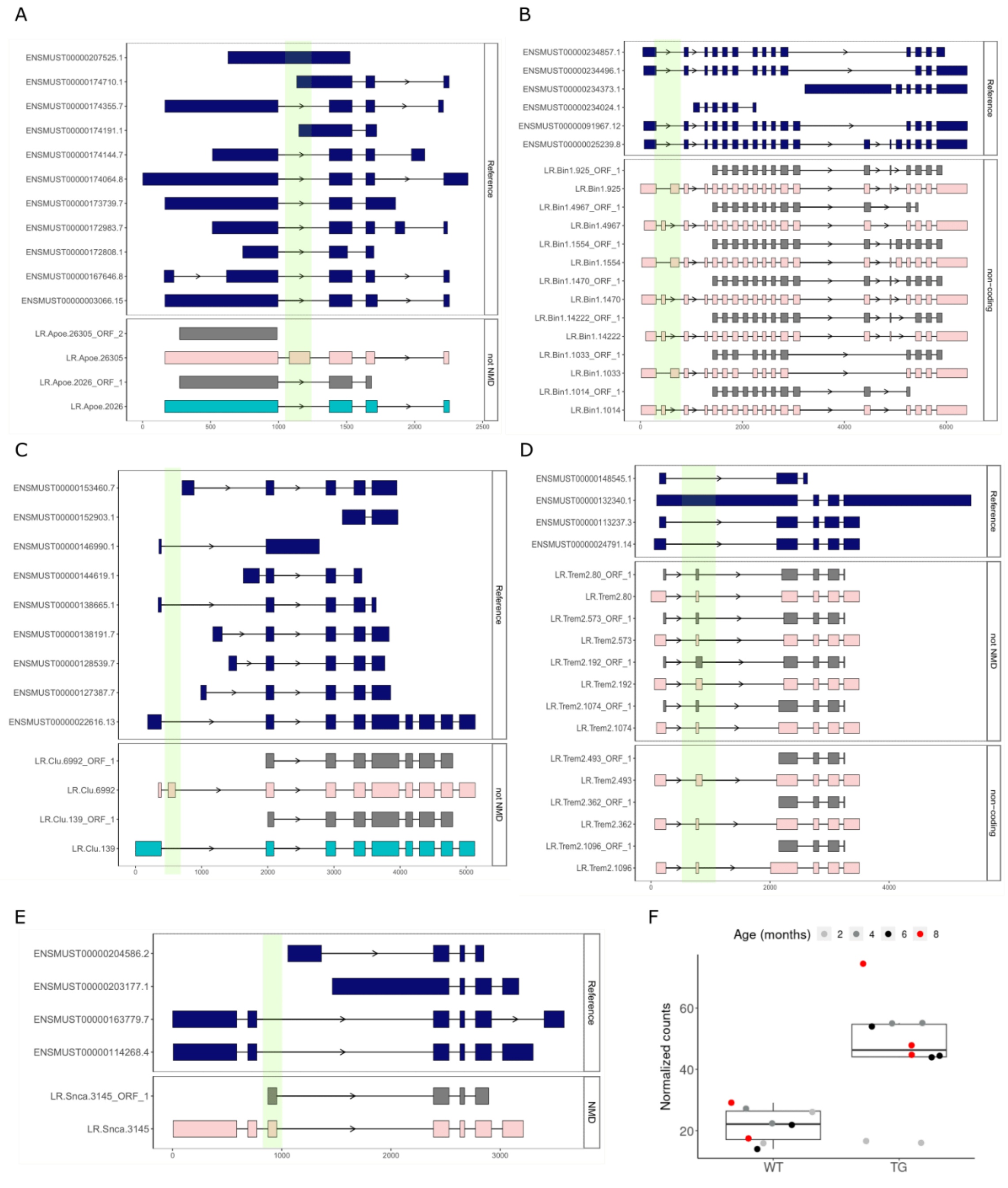

Supplementary Figure 10: Top-ranked differentially expressed transcripts between WT and TG mice.
Shown are box-plots of the **(A – J)** transcript expression of the 10 top-ranked differentially expressed transcripts between WT and TG mice (genotype). Transcript expression is determined from normalized ONT full length read count (Targeted ONT dataset). Differential transcript expression is performed using the Wald test in *DESeq2* (~ genotype). *SQANTI3* structural categories (FSM – Full Splice Match, ISM – Incomplete Splice Match, NIC **–** Novel in Catalog, NNC – Novel Not in Catalog) are provided in parenthesis for each transcript. WT – wild type, TG – transgenic.

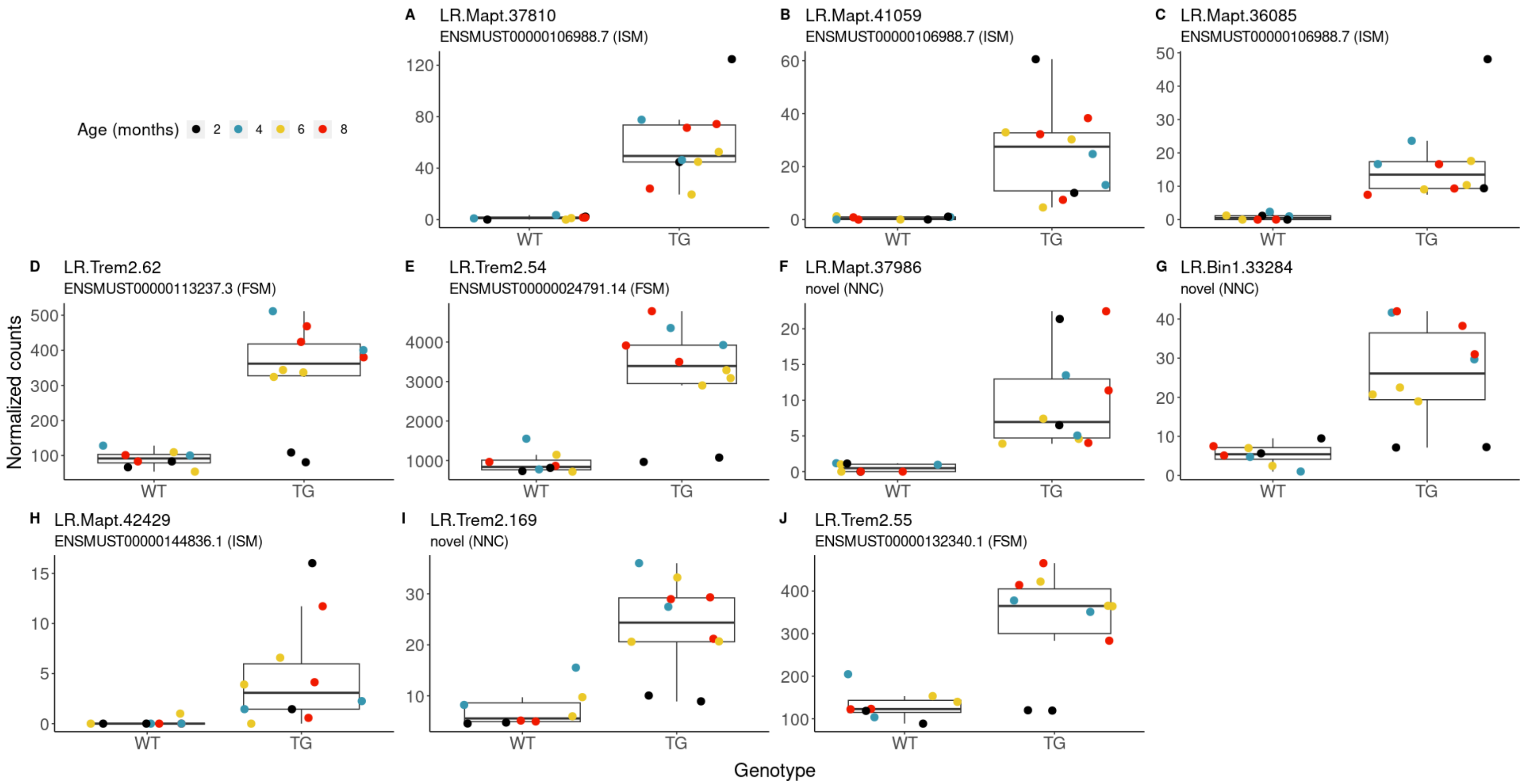

Supplementary Figure 11: Differential transcript usage
Shown are box plots of the transcript usage (isoform fraction) of the genes that were significantly altered **(A – C)** between WT (gray) and TG (black) mice and **(D – F)** associated with the progression of tau pathology in TG (black) mice. The usage of the top 3 most abundant transcripts are shown for each gene.

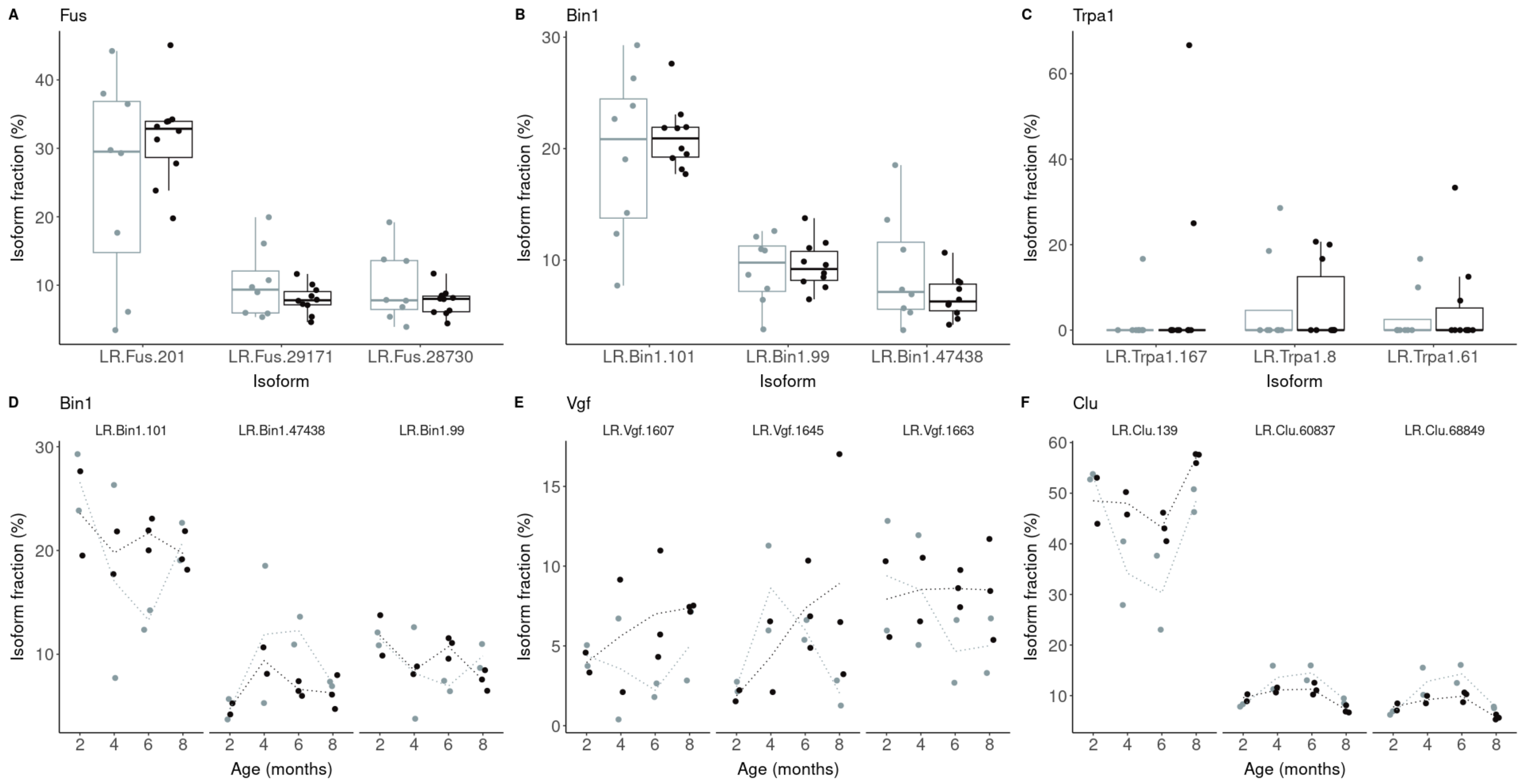

Supplementary Figure 12: Increase of *Trem2 and Cd33* IR-characterized transcripts in TG mice with progression of tau pathology.

Shown are **(A)** visualization tracks of *Trem2* transcripts characterized with intron retention events (IR), **(B)** box-plot of the total normalization counts of the shown *Trem2* IR-characterized transcripts summed between WT and TG mice and across age, **(C)** visualization tracks of *Cd33* transcripts with IR events, and **(D)** box-plot of the total normalization counts of the shown *Cd33* IR-characterized transcripts. A subset of the reference transcripts are also shown in blue. IR – Intron Retention.

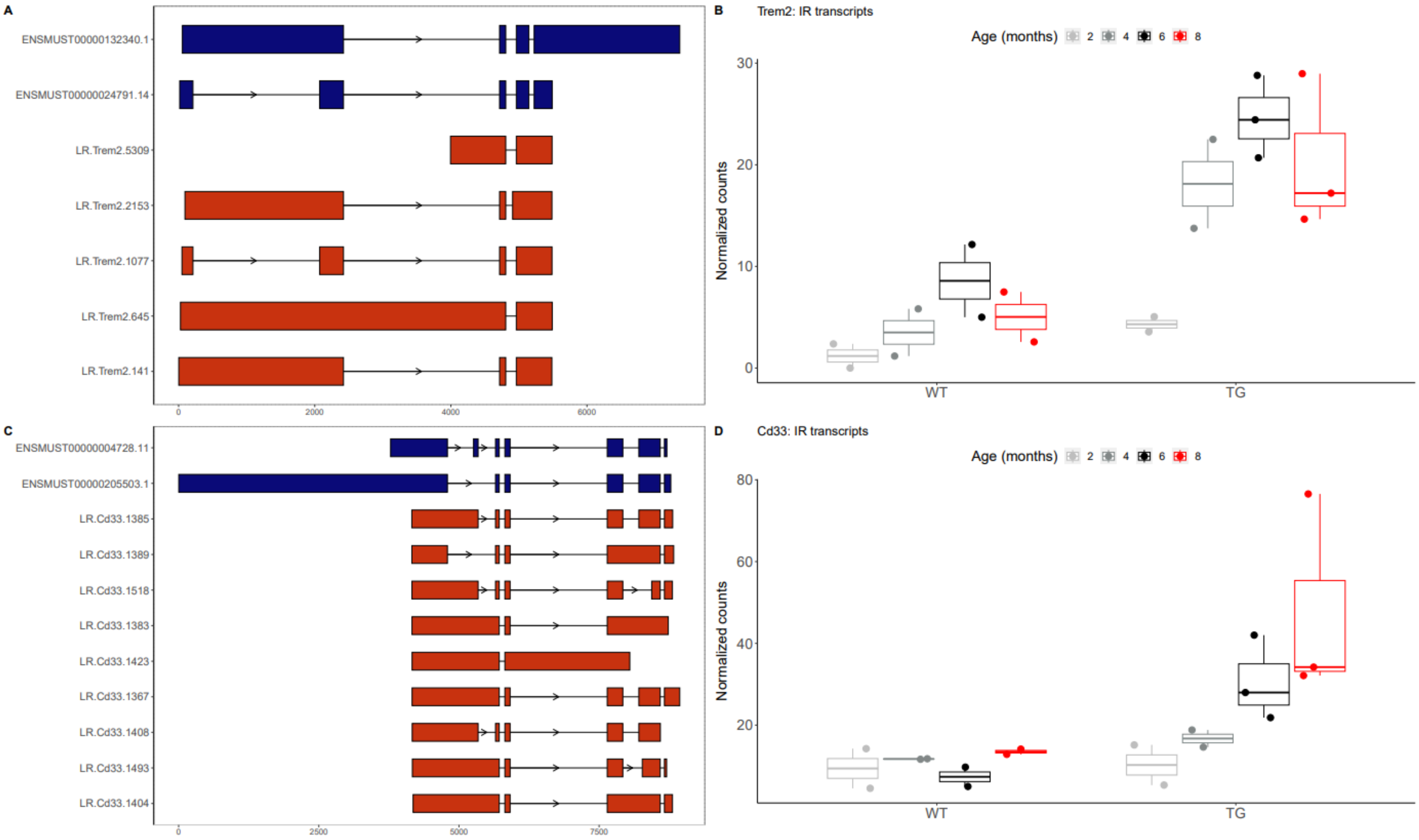

Supplementary Figure 13: Increase of *Trem2 and Cd33* NMD-predicted transcripts in TG mice.
Shown are **(A)** visualization tracks of *Trem2* transcripts predicted for nonsense-mediated decay, **(B)** box-plot of the total normalization counts of the shown *Trem2* NMD-predicted transcripts summed between WT and TG mice and across age, **(C)** visualization tracks of *Cd33* transcripts predicted for NMD, and **(D)** box-plot of the total normalization counts of the shown *Cd33* transcripts predicted for NMD. Of note, shown are the transcript structure (coloured by *SQANTI3* structural category: pink – NIC, red – NNC) and the best predicted open reading frame called from *CPAT*. A subset of the reference transcripts are also shown in blue. NMD – nonsense-mediated decay, NIC – Novel In Catalog, NNC – Novel Not in Catalog.

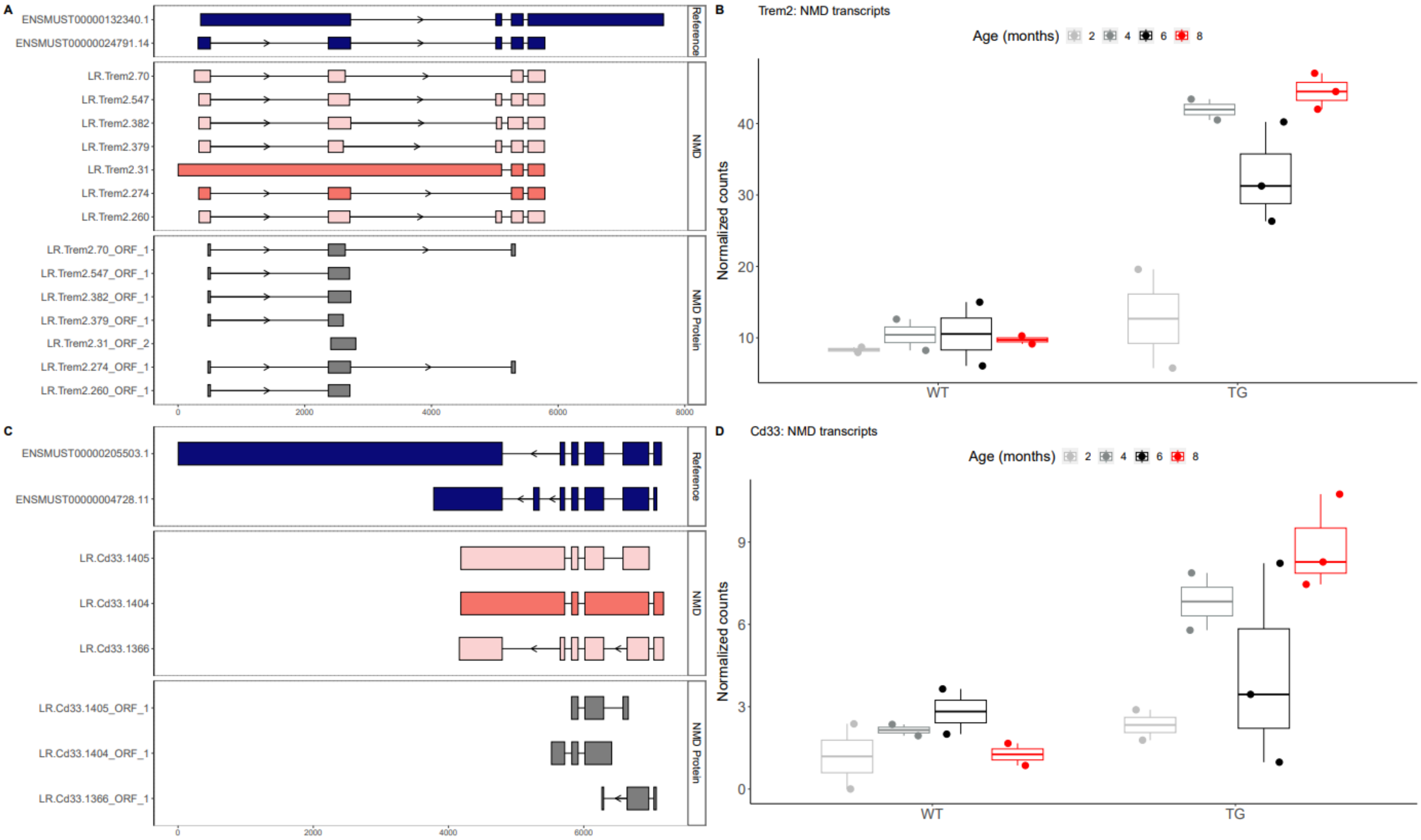

Supplementary Figure 14: Immunohistochemistry staining of Trem2 and Iba1 in rTg4510 mice.
Shown are representative immunohistochemistry images from staining Trem2 (red) and Iba1 (blue) in the hippocampus of WT and TG mice across 2, 4, 6 and 8 months.

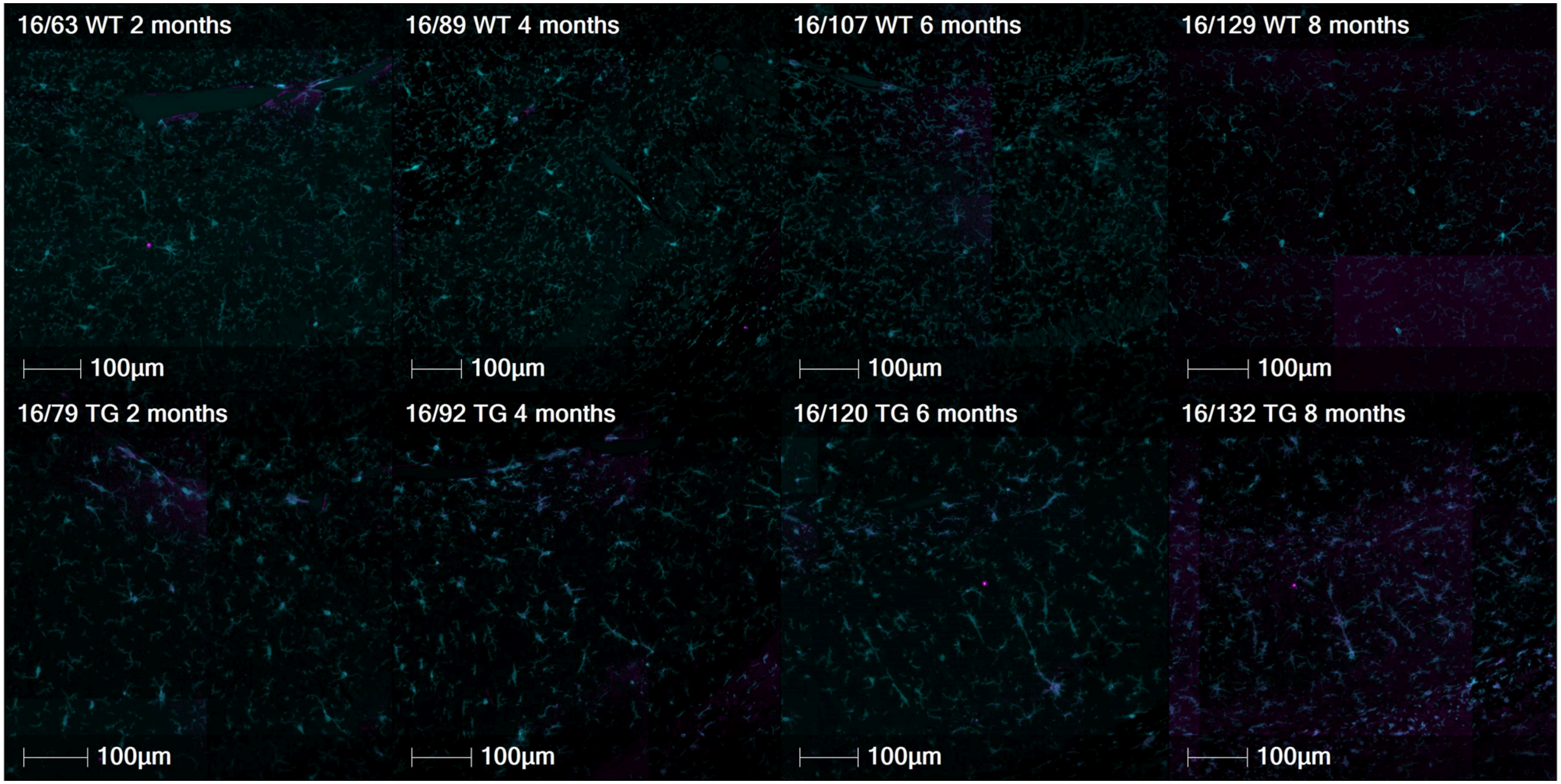

Supplementary Figure 15: Further characterization of *Trem2* in rTg4510 mouse model

(A) UCSC track of RNA-Seq data generated from matched samples, mouse reference transcripts (mm10, GENCODE) and Pfam domains of *Trem2*. The figure shows the variability of exon 2 (highlighted in red) – which encodes the V-set domain – confirmed by RNA-Seq data. **(B)** UCSC track of i) a selection of *Trem2*-associated isoforms, detected from targeted sequencing of rTg4510 mice, characterized with novel exons upstream of (highlighted in green) and within (highlighted in orange) the gene body, ii) the open reading frame (ORF; start is highlighted in blue) of shown *Trem2*-associated isoforms, showing that the novel upstream exon does not encode novel start exons whereas the internal novel exons (highlighted in orange) are retained within the ORF.

(A)

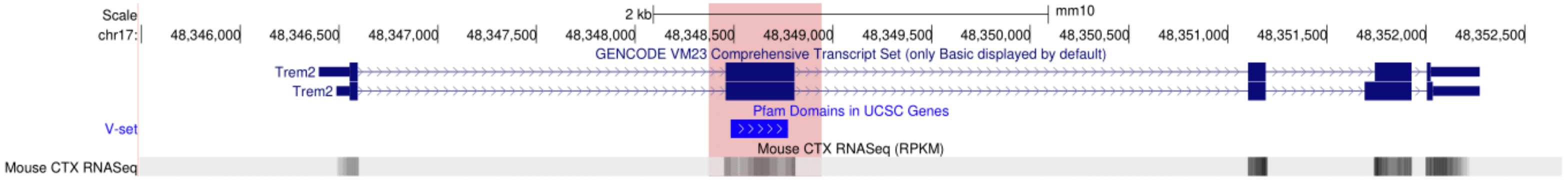

(B)

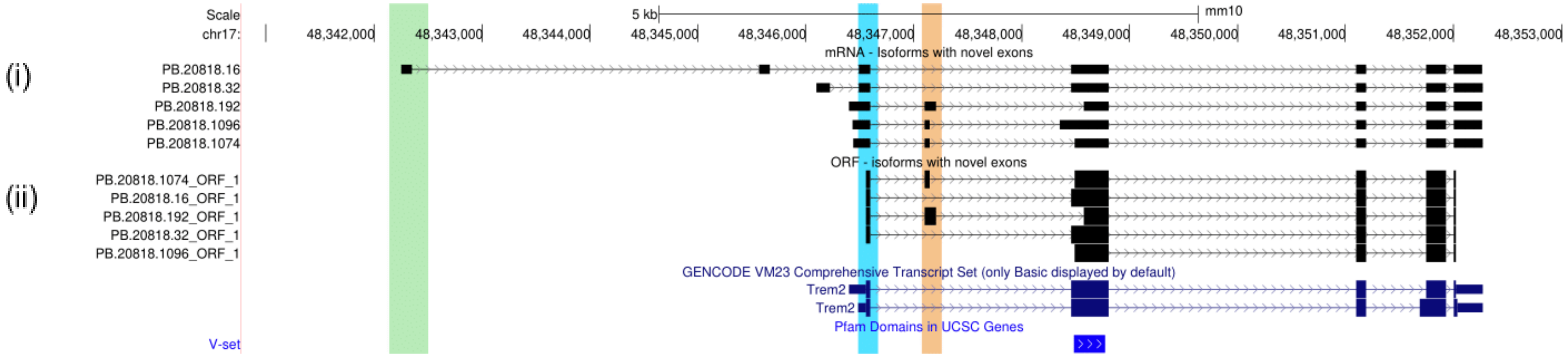

Supplementary Figure 16: Dominant *Clu/CLU* isoforms in rTg4510 and human AD cortex.

Shown are **(A)** visualization of the mouse dominant isoform (LR.Clu.139) and **(B)** its expression in WT and TG rTg4510 mice across age, **(C)** visualization of the homologous human dominant isoform (LR.CLU.402) and **(D)** its expression in AD brain (red) and controls (gray) across Braak stage. Normalized expression is deduced from normalizing ONT full-length read counts using *DESeq2*.

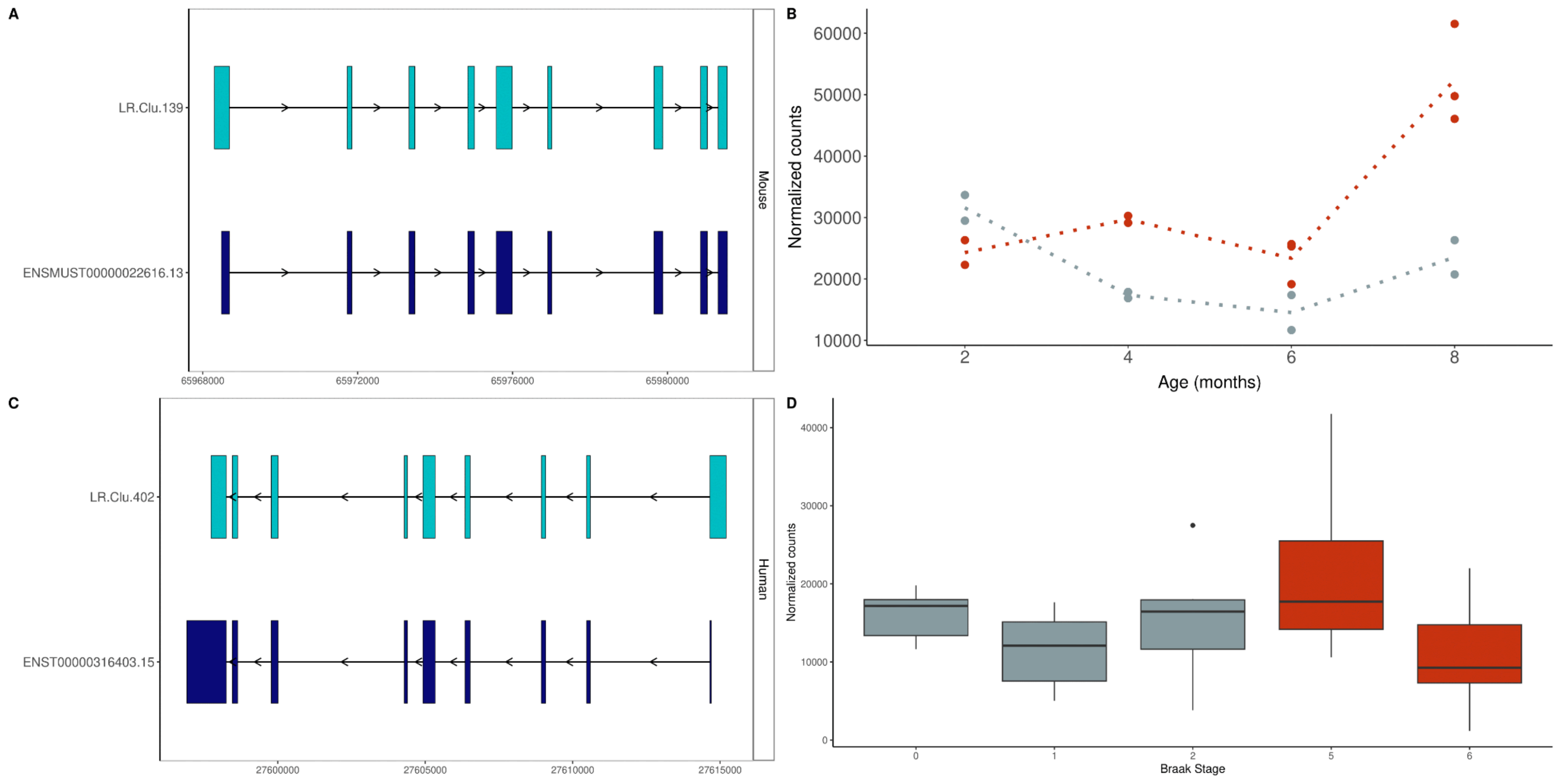

Supplementary Figure 17: Dominant *Bin1/BIN1* isoforms in rTg4510 and human AD cortex.
Shown are **(A)** visualization of the two dominant and differentially expressed isoforms (LR.Bin1.99, LR.Bin1.224) in rTg4510 mouse cortex. **(B)** LR.Bin1.224 normalized expression and **(C)** LR.Bin1.99 normalized expression in WT and TG rTg4510 mice across age. **(D)** Visualization of the respective homologous human dominant isoforms (LR.BIN1.13 homologous to mouse LR.Bin1.99), LR.BIN1.8 homologous to mouse LR.Bin1.224). **(E)** LR.BIN1.8 normalized expression and **(F)** LR.BIN1.13 normalized expression in AD brain (red) and controls (gray) across Braak stage. Normalized expression is deduced from normalizing ONT full-length read counts using *DESeq2*.

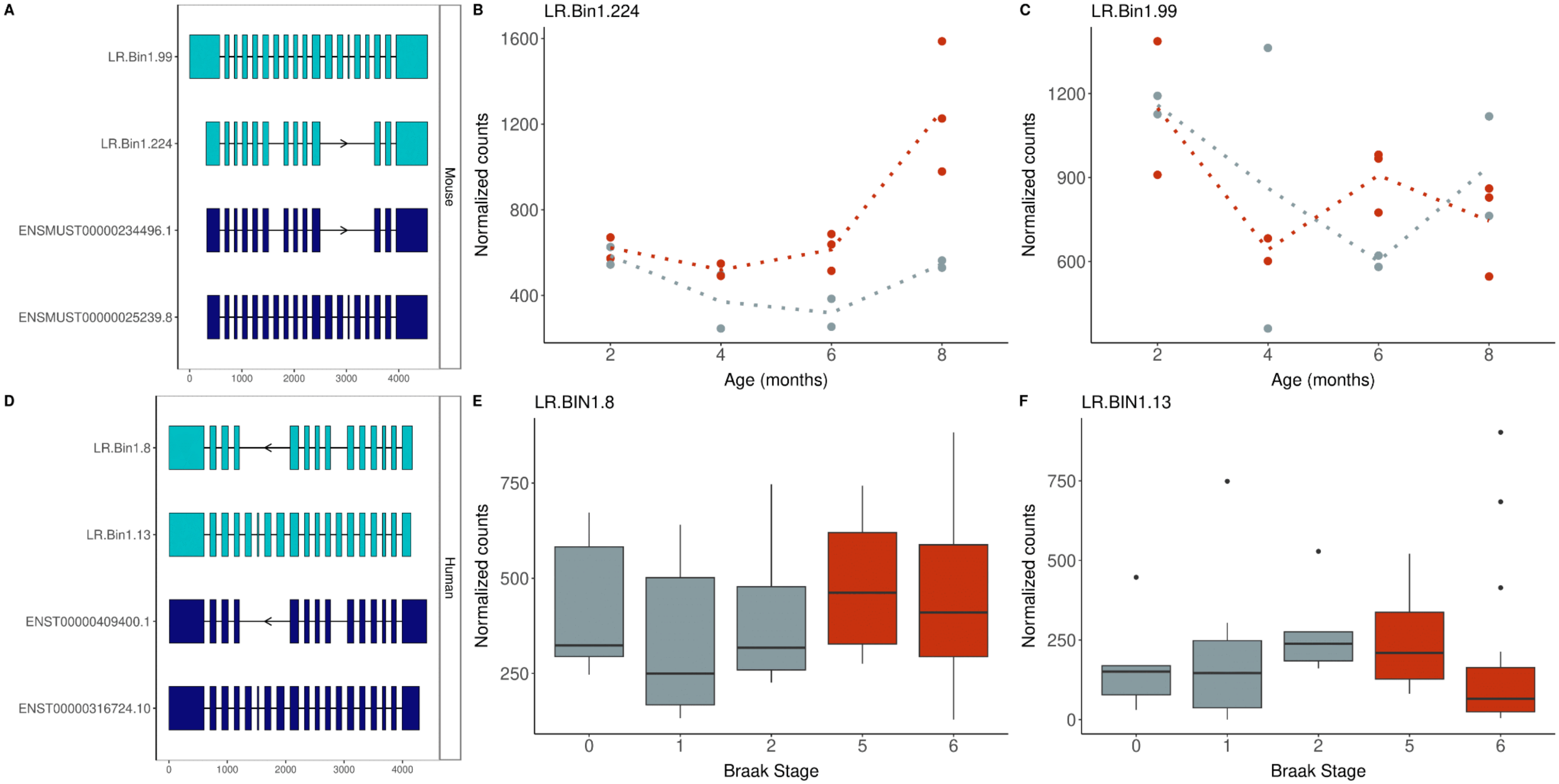

Supplementary Figure 18: FANS gating strategy

(A) Particles smaller than nuclei (black dots) were eliminated with an area plot of forward-scatter (FSC-A) versus side-scatter (SSC-A), with gating for nuclei-sized particles inside the gate (box). **(B)** Plots of height versus width in the side scatter channel are used for doublet discrimination with gating to exclude aggregates of two or more nuclei. **(C)** Doublet discrimination gating was used to isolate nuclei determined by sub-gating on Hoechst 33342. **(D, F)**Subsequent scatterplots discerning (d) NeuN-Alexa Fluor488–conjugated antibody staining (purple) **(E)** PU.1 PE-stained nuclei (dark pink) (f) the distribution of the three main nuclei subpopulations identified through double staining strategy (NeuN +ve, neurons; PU.1+ve, microglia, double–ve, oligodendrocytes enriched). The resultant hierarchical color key ensures that only nuclei that are negative for staining with the NeuN antibody are passed through the next gating condition. For more information on the FANS, please visit: https://www.protocols.io/view/fluorescence-activated-nuclei-sorting-fans-of-puri-dm6gpbwndlzp/v1

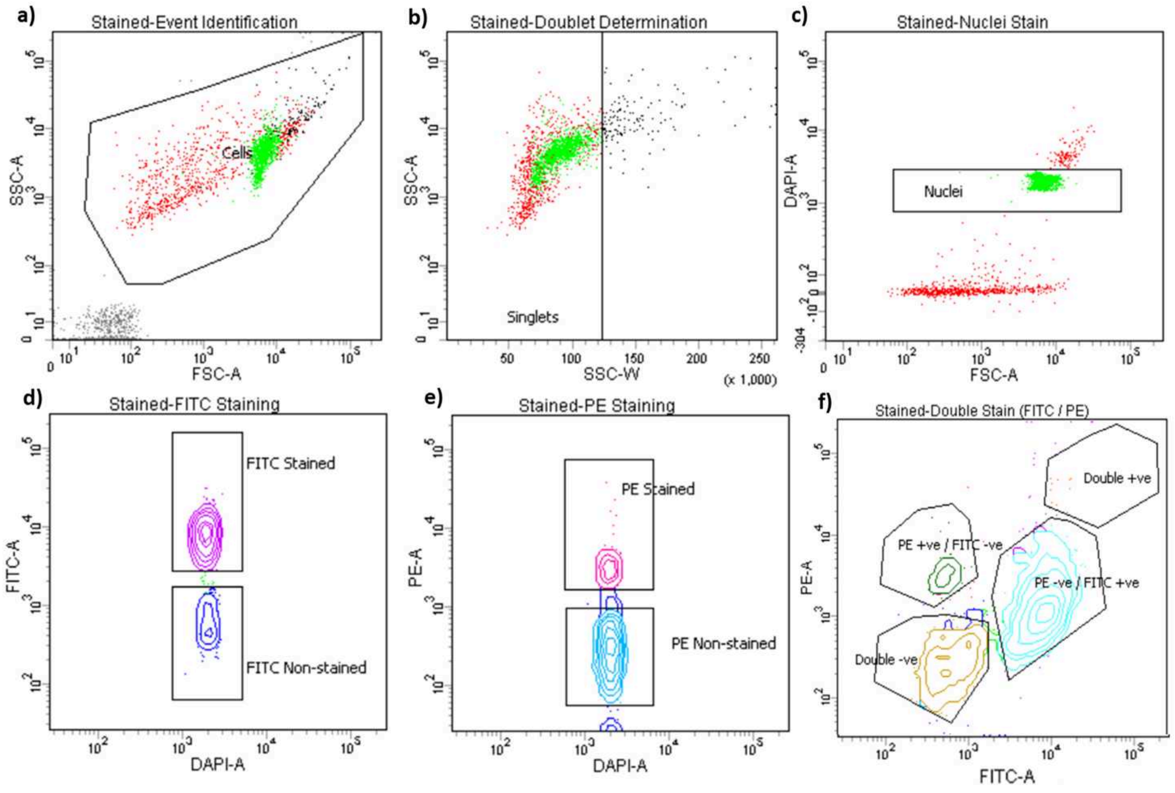

Tube: Stained

Population	#Events	%Parent	%Total
All Events	3,000	####	100.0
Cells	2,165	72.2	72.2
Singlets	2,027	93.6	67.6
Nuclei	1,245	61.4	41.5
FITC Non-stained	325	26.1	10.8
PE Stained	60	18.5	2.0
PE Non-stained	228	70.2	7.6
FITC Stained	910	73.1	30.3
PE +ve / FITC -ve	53	4.3	1.8
Double +ve	11	0.9	0.4
Double -ve	227	18.2	7.6
PE -ve / FITC +ve	872	70.0	29.1

Supplementary Figure 19: Uncropped scan from immunohistochemistry staining

Shown is an uncropped image from immunohistochemistry staining of hippocampal tissue from rTg4510 TG mice at 8 months with Iba1 (cyan), Trem2 (red) and AT8Tau (green) staining. The area boxed in red corresponds to the zoomed-in figure shown in Figure 5E.

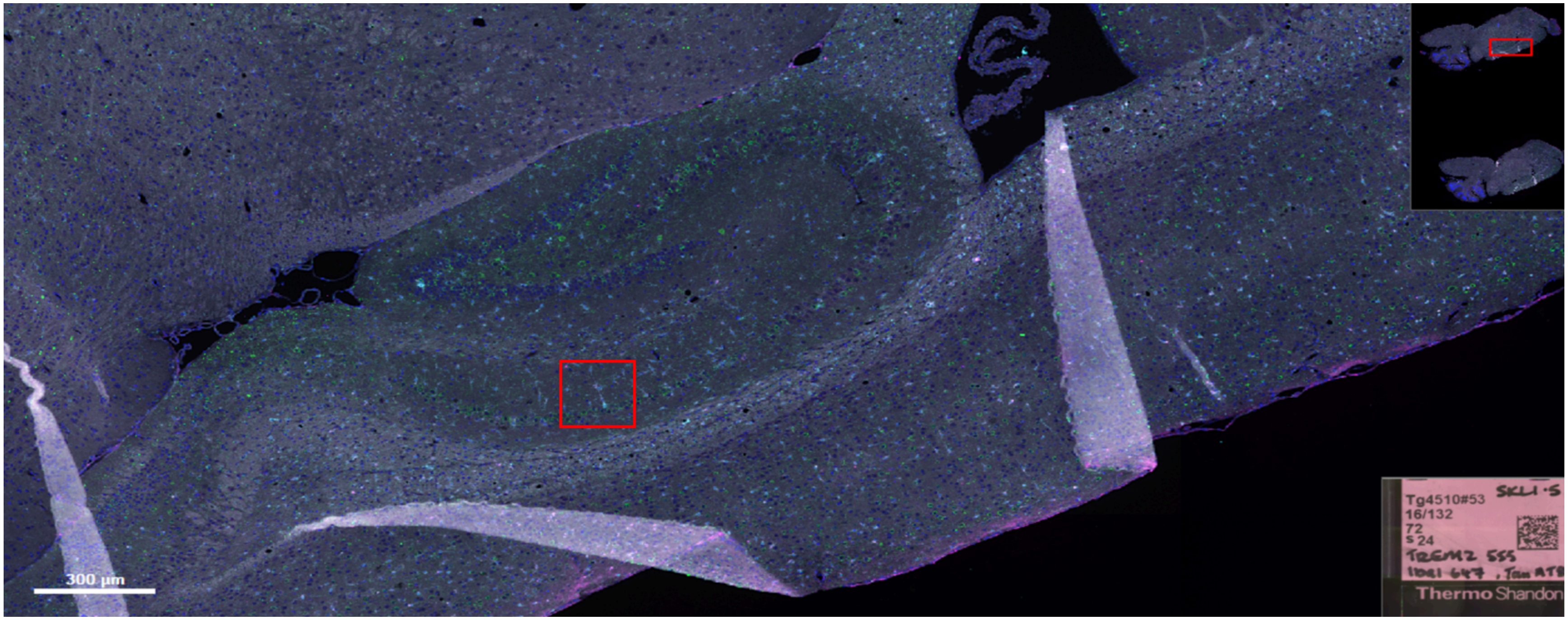

Supplement: Supplementary file 1 — Supplementary Information [file 41467_2024_50486_MOESM1_ESM.pdf]
