# Supplementary material for: Long-read transcript sequencing identifies differential isoform expression in the entorhinal cortex in a transgenic model of tau pathology
Source: Nat Commun. 2024 Aug 2;15:6458. doi: 10.1038/s41467-024-50486-8 (PMC11297290; doi:10.1038/s41467-024-50486-8)
Supplement: Supplementary file 3 — Description of Additional Supplementary Files [file 41467_2024_50486_MOESM3_ESM.pdf]

Description of Additional Supplementary Files

Supplementary Data 1: Phenotypic information for the mouse samples sequenced in this study. "X" denotes the samples that were sequenced per dataset and profiled for immunohistochemistry. FANS – Fluorescence-activated nuclei sorting, IHC – Immunohistochemistry, TG – rTg4510 transgenic mice, WT – Wild-type mice.

Supplementary Data 2: Isoform diversity in rTg4510 mouse model between genotype and age from whole transcriptome PacBio Iso-Seq dataset. FSM – Full Splice Match, ISM – Incomplete Splice Match, NIC – Novel in Catalog, NNC – Novel Not in Catalog.

Supplementary Data 3: Differentially expressed genes associated with the progression of tau pathology in the whole transcriptome PacBio Iso-Seq dataset. Gene expression is determined from the summation of normalized Iso-Seq full-length read counts (whole transcriptome dataset) of associated transcripts. Differential gene expression is performed using the Wald test in *DESeq2* (~ genotype + age + genotype * age). The counts of the genotype groups refer to the mean normalized full-length reads. TG – rTg4510 transgenic mice, WT – Wild-type mice.

Supplementary Data 4: Differentially expressed transcripts associated with the progression of tau pathology in the whole transcriptome PacBio Iso-Seq dataset. Transcript expression is derived from normalized Iso-Seq full-length read counts (whole transcriptome dataset). Differential transcript expression is performed using the Wald test in *DESeq2* (~ genotype + age + genotype * age). log2FC – $\log_2$ fold-change, mos – months, TG – rTg4510 transgenic mice, WT – Wild-type mice.

Supplementary Data 5: Protein identification in targeted transcriptome. AD-associated proteins predicted from using an established long-read proteogenomics pipeline.

Supplementary Data 6: Differentially expressed transcripts between WT and rTg4510 mice across age (pathology) in targeted ONT and Iso-Seq datasets. Transcript expression is derived from normalized ONT and Iso-Seq full-length read counts (ONT and Iso-Seq targeted datasets). Differential transcript expression analysis was performed using the Wald test in *DESeq2* (~ genotype + age + genotype * age) across all time points in ONT and Iso-Seq targeted datasets. *SQANTI3* structural categories (FSM – Full Splice Match, ISM – Incomplete Splice Match, NIC – Novel in Catalog, NNC – Novel Not in Catalog) and subcategories are provided for each transcript. WT – wild type, TG – transgenic. Significant transcripts (FDR < 0.05) are highlighted in green. Direction of effect refers to the transcript abundance in TG mice relative to WT mice.

Supplementary Data 7: Differentially expressed transcripts between WT and rTg4510 mice (genotype) in the targeted ONT and Iso-Seq datasets. Transcript expression is derived from normalised ONT and Iso-Seq full-length read counts (ONT and Iso-Seq targeted datasets). Differential transcript expression analysis was performed using the Wald test in *DESeq2* (~ genotype) between WT and TG mice in ONT and Iso-Seq targeted datasets. *SQANTI3* structural categories (FSM – Full Splice Match, ISM – Incomplete Splice Match, NIC – Novel in Catalog, NNC – Novel Not in Catalog) and subcategories are provided for each transcript. WT – wild type, TG – transgenic. Significant transcripts (FDR < 0.05) are highlighted in green. Direction of effect refers to the transcript abundance in TG mice relative to WT mice.

Supplementary Data 8: Genes with differential transcript usage between WT and rTg4510 mice in targeted dataset. Transcript expression is derived from the normalized ONT full-length read counts (ONT targeted datasets). Differential transcript usage between experimental groups (Genotype: WT and TG, Pathology: WT and TG across 4 time points) was performed using *EdgeR spliceVariants* with filtering of minor isoforms using fold-change (log2) (see Methods). A "TRUE" podium change indicates a switch in the major isoform usage (i.e. switch of the dominant most abundant isoform).

Supplementary Data 9: Phenotypic information for the AD post-mortem brain samples sequenced in this study.

Supplementary Data 10: Isoform diversity of AD-associated genes in AD human cortex. AD-associated transcripts detected from targeted ONT sequencing in post-mortem cortex tissue dissected from 44 individuals (21 individuals with advanced AD neuropathology and 23 neuropathology-free control donors).

Supplementary Data 11: Read coverage per mouse sample in whole and targeted datasets. Tabulated are the total number of reads sequenced per sample using **(A)** Iso-Seq (PacBio) whole transcriptome (n = 12 samples, 6 WT and 6 TG), **(B)** Iso-Seq targeted transcriptome (n = 24 samples, 12 WT and 12 TG) and ONT targeted transcriptome (n = 18 samples, 8 WT and 10 TG). The read counts are deduced from the number of raw reads retained after demultiplexing and removing primers/barcode sequences.

Supplementary Data 12: Primers and barcodes used for cDNA synthesis. Sequences used for cDNA synthesis. SMARTer PCR Oligo and PolyT blocker Oligo were needed for First strand cDNA synthesis (Clontech, UK). Barcodes 1 – 10, designed by PacBio, were used for multiplexing in targeted experiments. All sequences were ordered through IDT under PAGE purification. Oligo – Oligonucleotide.

Supplementary Data 13: Custom-designed mouse probes for AD target genes. Mouse probes (IDT, mm10) were designed to enrich 20 AD-associated genes for targeted sequencing of rTg4510 WT and TG mice (n = 24).

Supplementary Data 14: Custom-designed human probes for AD target genes. Human probes (IDT, hg19) were designed to enrich 20 AD-associated genes for targeted sequencing of human post-mortem brain tissue (n = 44)
