# Supplementary material for: Long-read transcript sequencing identifies differential isoform expression in the entorhinal cortex in a transgenic model of tau pathology
Source: Nat Commun. 2024 Aug 2;15:6458. doi: 10.1038/s41467-024-50486-8 (PMC11297290; doi:10.1038/s41467-024-50486-8)
Supplement: Supplementary file 18 — Reporting Summary [file 41467_2024_50486_MOESM18_ESM.pdf]

Corresponding author(s): Jonathan Mill, Szi Kay Leung

Last updated by author(s): 26/06/24

Reporting Summary

Nature Portfolio wishes to improve the reproducibility of the work that we publish. This form provides structure for consistency and transparency in reporting. For further information on Nature Portfolio policies, see our Editorial Policies and the Editorial Policy Checklist.

Statistics

For all statistical analyses, confirm that the following items are present in the figure legend, table legend, main text, or Methods section.

n/a	Confirmed	
☐	☒	The exact sample size (*n*) for each experimental group/condition, given as a discrete number and unit of measurement
☐	☒	A statement on whether measurements were taken from distinct samples or whether the same sample was measured repeatedly
☐	☒	The statistical test(s) used AND whether they are one- or two-sided<br>*Only common tests should be described solely by name; describe more complex techniques in the Methods section.*
☐	☒	A description of all covariates tested
☐	☒	A description of any assumptions or corrections, such as tests of normality and adjustment for multiple comparisons
☐	☒	A full description of the statistical parameters including central tendency (e.g. means) or other basic estimates (e.g. regression coefficient) AND variation (e.g. standard deviation) or associated estimates of uncertainty (e.g. confidence intervals)
☐	☒	For null hypothesis testing, the test statistic (e.g. *F*, *t*, *r*) with confidence intervals, effect sizes, degrees of freedom and *P* value noted<br>*Give P values as exact values whenever suitable.*
☒	☐	For Bayesian analysis, information on the choice of priors and Markov chain Monte Carlo settings
☒	☐	For hierarchical and complex designs, identification of the appropriate level for tests and full reporting of outcomes
☐	☒	Estimates of effect sizes (e.g. Cohen's *d*, Pearson's *r*), indicating how they were calculated

Our web collection on statistics for biologists contains articles on many of the points above.

Software and code

Policy information about availability of computer code

Data collection

Long-read cDNA sequencing data was generated from mouse and human entorhinal cortex tissue. No software or code was used in the collection of these data

Data analysis

Iso-Seq raw read processing: Iso-Seq v3
ONT raw read base-calling: Guppy v4.0
Iso-Seq barcode and primer demultiplex: Lima v2.0
ONT barcode and primer demultiplex: Porechop v0.2.4
ONT polyA removal: Cutadapt (v2.9)
Alignment: Minimap2 v2.1.7, pbmm2 v10.0
ONT read correction: TranscriptClean v.2.1
Transcript collapse: Iso-Seq collapse v3.8.2
Transcript annotation: SQANTI3 v.5.0
Isoform characterization: FICLE v1.1.2
Isoform visualisation: ggtranscript v.0.99.9
Differential expression analysis: DESeq2 v.1.26.0
Differential usage analysis: EdgeR spliceVariant v.3.28.1, tappAS v.1.0.7
Long-read proteogenomics pipeline v1.0.0
ORF calling: CPAT v.3.0.2
Immunohistochemistry slide analysis: HALO sofware v3.6

All original code supporting this study is available at https://github.com/SziKayLeung/rTg451048 (doi:10.5281/ZENODO.12191598), and the

FICLE package is available to download at https://github.com/SziKayLeung/FICLE. Detailed protocols for FANS and our modified ONT library preparation approach are available on protocols.io (https://doi.org/10.17504/protocols.io.dm6gpbwndlzp/v1; https://doi.org/10.17504/protocols.io.kqdg3xzwzg25/v).

For manuscripts utilizing custom algorithms or software that are central to the research but not yet described in published literature, software must be made available to editors and reviewers. We strongly encourage code deposition in a community repository (e.g. GitHub). See the Nature Portfolio guidelines for submitting code & software for further information.

Data

Policy information about availability of data

All manuscripts must include a data availability statement. This statement should provide the following information, where applicable:

- Accession codes, unique identifiers, or web links for publicly available datasets
- A description of any restrictions on data availability
- For clinical datasets or third party data, please ensure that the statement adheres to our policy

Raw ONT and PacBio Iso-Seq data generated in this study have been deposited in the Sequence Read Archive (SRA) database (https://www.ncbi.nlm.nih.gov/sra) under accession numbers PRJNA981131 (rTg4510 targeted ONT and PacBio Iso-Seq data), PRJNA663877 (rTg4510 whole transcriptome Iso-Seq data) and PRJNA1085642 (AD post-mortem brain targeted ONT data). The processed intermediate data and UCSC genome browser tracks (merged mouse targeted data, mouse whole transcriptome Iso-Seq data, mouse sorted data and human AD targeted data) are available for download at: https://zenodo.org/doi/10.5281/zenodo.8101907.

Research involving human participants, their data, or biological material

Policy information about studies with human participants or human data. See also policy information about sex, gender (identity/presentation), and sexual orientation and race, ethnicity and racism.

Reporting on sex and gender	Females = 22, Males = 22. This is reported in Supplementary Table 9.<br>Because we used tissue from an equal number of male and female donors and our sample size was not powered to differentiate sex-effects, we did not include sex as a covariate in our analyses of human tissue.
Reporting on race, ethnicity, or other socially relevant groupings	NA.
Population characteristics	Post-mortem human cortex samples from 44 individuals (21 with advanced AD neuropathology and 23 with minimal neuropathology) were provided by the Brains for Dementia Research (BDR) Cohort.<br>Mean Age (SD) = 81.41 (9.3)<br>Females = 22, Males = 22<br>Mean Braak NFT (neurofibrillary tangles) stage (SD) = 3.3 (2.5)
Recruitment	BDR participants were recruited using both national and local press (e.g. newspapers, newsletters, leaflets), TV and radio coverage as well as at memory clinics and support groups.
Ethics oversight	Ethical approval for the study was granted and approved by the University of Exeter Medical School Research Ethics Committee (13/02/009).

Note that full information on the approval of the study protocol must also be provided in the manuscript.

Field-specific reporting

Please select the one below that is the best fit for your research. If you are not sure, read the appropriate sections before making your selection.

☒ Life sciences ☐ Behavioural & social sciences ☐ Ecological, evolutionary & environmental sciences

For a reference copy of the document with all sections, see nature.com/documents/nr-reporting-summary-flat.pdf

Life sciences study design

All studies must disclose on these points even when the disclosure is negative.

Sample size	In total we performed long-read cDNA sequencing (PacBio and ONT) in dissected entorhinal cortex tissue from 12 independent female rTg4510 transgenic (TG) mice and 12 independent female wild-type (WT) mice, across 4 different time points (2, 4, 6 and 8 months). This cohort represents a subset of dissected entorhinal cortex tissue from 59 mice (30 TG and 29 WT) previously sequenced using RNA-Seq (Illumina) (Castanho et al. 2020). We also performed targeted ONT sequencing in post-mortem human cortex samples from individuals with and without advanced AD neuropathology (n = 21 AD, n = 23 controls).
Data exclusions	No mouse samples from the whole transcriptome and targeted datasets were excluded from analysis. However, we applied a stringent QC pipeline: (1) raw ONT reads with Phred (Q) < 7 were discarded, (2) only full-length raw ONT and Iso-Seq reads with correct barcode and primer orientations and combinations were retained, (3) after alignment to the mouse reference genome, only transcripts with 95% alignment identity and 85% coverage were retained for Iso-Seq collapse, (4) transcripts were further annotated and filtered using SQANTI to remove

technical artifacts (from intrapriming and template-switching during cDNA synthesis), (5) finally, we applied an expression threshold (minimum 10 reads across any 5 samples) to filter for rare transcripts.
Three human samples (sample 12, 16, 27) were excluded from differential expression analysis due to low sequencing depth.

Replication	Our samples were sequenced using five complementary approaches: (1) ultra-deep targeted nanopore ONT cDNA sequencing of 20 target genes, (2) whole transcriptome and targeted PacBio Iso-Seq of the same panel of genes, (3) fluorescence-activated nuclei sorting (FANS) followed by ONT nanopore sequencing to explore the extent to which DETs associated with tau pathology are expressed in specific cell populations, (4) short-read RNA-Seq data (Castanho et al. 2020) to validate splice junctions and transcript annotations, (5) immunohistochemistry analyses to show that the upregulation of Trem2 (driven by increased expression of the most abundant transcript) co-localises with tau pathology in microglia.
Randomization	Samples were randomized at all experimental stages prior to sequencing, with processing batches comprising an equal number of WT and TG mouse samples, to avoid bias and technical batch effects. All human post-mortem samples were barcoded and sequenced in one flow cell to avoid batch effects.
Blinding	Samples were labeled with anonymized ID codes and processed in batches, blinding disease status from the experimenter/analyst for individual samples.

Reporting for specific materials, systems and methods

We require information from authors about some types of materials, experimental systems and methods used in many studies. Here, indicate whether each material, system or method listed is relevant to your study. If you are not sure if a list item applies to your research, read the appropriate section before selecting a response.

Materials & experimental systems

n/a	Involved in the study	
☐	☒	Antibodies
☒	☐	Eukaryotic cell lines
☒	☐	Palaeontology and archaeology
☐	☒	Animals and other organisms
☒	☐	Clinical data
☒	☐	Dual use research of concern
☒	☐	Plants

Methods

n/a	Involved in the study	
☒	☐	ChIP-seq
☐	☒	Flow cytometry
☒	☐	MRI-based neuroimaging

Antibodies

Antibodies used	Immunohistochemistry:<br>Primary for microglia: Rabbit recombinant monoclonal Iba1 antibody (ab221790, Abcam) at a 1:500 dilution<br>Secondary for microglia: Goat anti-rabbit IgG secondary antibody, Alexa Fluor 647 (A21245, Invitrogen) at a 1:400 dilution<br><br>Primary for Trem2: Rabbit recombinant monoclonal TREM2 antibody (ab305103, Abcam) at a 1:500 dilution incubated overnight at 4°C<br>Secondary for Trem2: Alexa Fluor 555 Tyramide SuperBoost™ Kit, goat anti-rabbit IgG (B40923, Invitrogen) according to manufacturer's instructions<br><br>Primary for tau: Phospho Tau (Ser202, Thr205) monoclonal Antibody (AT8) antibody (Thermo Fisher Scientific, MN1020) at a 1:500 dilution overnight at at 4°C<br>Secondary for tau: Goat anti-mouse 488 (A10680, Invitrogen) as the secondary antibody at 1:400 dilution<br><br>FANS:<br>NeuN marker: Rabbit monoclonal NeuN antibody conjugated to Alexa Fluor 488 (ab190195, Abcam) at a 1:1000 dilution
Validation	All antibodies used are pre-conjugated and prevalidated by the manufacturers and extensive work in our lab.

Animals and other research organisms

Policy information about studies involving animals; ARRIVE guidelines recommended for reporting animal research, and Sex and Gender in Research

Laboratory animals	rTg4510 (rTg(tet-o-TauP301L)4510), licensed from the Mayo Clinic (Jacksonville, FL, USA), were bred on a mixed 129S6/SvEvTac + FVB/NCrl background (heterozygous tau responder x heterozygous tTA effector). Bi-transgenic (CC, here referred as TG) female mice and littermate controls (WW, here identified as WT) at ages 2, 4, 6 and 8 months old were used for this study. All mice were bred and delivered to Eli Lilly and Company (Windlesham, UK) by Envigo (Loughborough, UK). At Eli Lilly, animals were housed under standard conditions (constant temperature and humidity) with a 12h light/dark cycle in individually ventilated cages (up to 5 animals per cage), with free access to food (Teklad irradiated global rodent diet (Envigo, United Kingdom)) and water.
Wild animals	This study did not involve wild animals.

Reporting on sex	Only female mice were used in this study because of their elevated and more progressive pathology compared to males, and to minimize heterogeneity between samples.
Field-collected samples	This study did not involve samples collected from the field.
Ethics oversight	Mouse entorhinal cortex tissue was dissected from female WT and rTg4510 TG age-matched littermate mice in accordance with the UK Animals (Scientific Procedures) Act 1986 and with approval of the local Animal Welfare and Ethical Review Board.

Note that full information on the approval of the study protocol must also be provided in the manuscript.

Flow Cytometry

Plots

Confirm that:

☐ The axis labels state the marker and fluorochrome used (e.g. CD4-FITC).

☒ The axis scales are clearly visible. Include numbers along axes only for bottom left plot of group (a 'group' is an analysis of identical markers).

☒ All plots are contour plots with outliers or pseudocolor plots.

☒ A numerical value for number of cells or percentage (with statistics) is provided.

Methodology

Sample preparation	Neuron-enriched and glia-enriched nuclei populations were isolated from cortex dissected from a subset of 8 mice (4 WT and 4 TG at ages 2 and 8 months) using the nuclear marker NeuN.
Instrument	FACS Aria III cell sorter
Software	A full protocol detailing each step of our nuclei purification protocol is provided at protocols.io (https://www.protocols.io/view/fluorescence-activated-nuclei-sorting-fans-of-puri-b4ehqtb6; dx.doi.org/10.17504/protocols.io.dm6gpbwndlzp/v1).
Cell population abundance	Our protocol yields ~60,000 NeuN+ve (neuron enriched) and ~20,000 NeuN-ve (oligodendrocyte enriched) nuclei per ≤ 100 mg of frozen mouse cortex tissue. Recovery might vary from sample to sample due to high inter-sample variability and regional differences across cortical areas (i.e. dissection procedure, fat content of tissue sectioned, age).
Gating strategy	Stained and unstained tubes for each sample were loaded individually for data acquisition. A preliminary qualitative analysis of the data acquired was essential to select the appropriate gating strategy to maximize the nuclei capture while excluding unnecessary debris and to ensure optimal signal/noise ratio.<br><br>Gating Parameters (X-axis: Y-axis):<br>FSC-A:SSC-A (Size, cell granularity or internal complexity)<br>SSC-W:SSC-A (to gate out doublets)<br>FSC-A:DAPI-A (to gate the single nuclei population)<br>DAPI-A:FITC-A (to gate NeuN stained nuclei)<br>DAPI-A:PE-A (to gate PU.1 stained nuclei)<br>FITC-A: PE-A (to visualize the distribution of the three detectable populations)

☒ Tick this box to confirm that a figure exemplifying the gating strategy is provided in the Supplementary Information.
